# Editorial: Floral biology: understanding and applications

**DOI:** 10.3389/fpls.2024.1449153

**Published:** 2024-06-26

**Authors:** Amy L. Klocko, Tomoya Esumi, P. William Hughes

**Affiliations:** ^1^ Department of Biology, University of Colorado Colorado Springs, Colorado Springs, CO, United States; ^2^ Academic Assembly Institute of Agricultural and Life Sciences, Shimane University, Matsue, Japan; ^3^ Department of Ecology, Environment, and Plant Sciences, Stockholm University, Stockholm, Sweden

**Keywords:** floral biology, flowering locus T (FT), crops, agriculture, floral transition, floral structure, floral longevity

Flowering plants and their fruit and seed products are a foundational component in agricultural systems. Flowers are also a valuable horticultural trait, with many ornamental species grown specifically for their showy blooms. Much of what we have learned of the foundational basis of floral timing and formation has been gained from studies using the model species *Arabidopsis thaliana* (*Arabidopsis*) (Coen and Meyerowitz, 1991; Causier et al., 2010). As a result, many genes are now known to be conserved across a wide variety of agriculturally and horticulturally important species with widely diverse floral forms (Rehman et al., 2023). At present, researchers seek to apply current knowledge to other species of interest and to make new discoveries in existing systems. This research topic showcases important new findings in the area of floral biology.

One key historical discovery in floral biology was the identification of *FLOWERING LOCUS T* (*FT*), a main inducer of the transition from vegetative to reproductive growth (Kobayashi et al., 1999). Current research aims to elucidate the contributions of *FT* homologs and *FT*-associated genes, including the repressor *FLOWERING LOCUS C* (*FLC*) and the modulators *FLOWERING LOCUS D* (*FD*), *CONSTANS* (*CO*), and *GIGANTEA* (*GI*), to floral onset in species with different light requirements (Kim, 2020; Rehman et al., 2023). In Lee et al. the authors review *FT* gene family expression in long-day, short-day, and day neutral crops. In many species, floral onset is a desirable outcome, while in root crops such as *Beta vulgaris* (beet) and leafy crops including *Lactuca sativa* (lettuce) and *Spinacia oleracea* (spinach), delayed onset can lead to improved crop quality and yield. Moreover, understanding which *FT*-like genes act as floral activators or repressors, as well as how each gene is expressed under different light conditions, is valuable information that can be used to optimize the timing of floral onset. Furthermore, *FT* can also be utilized as a tool for shortening the juvenility of woody perennials using genetic transformation. Using such a procedure, the induction of precocious flowering in fruit tree species can accelerate associated breeding programs to a considerable degree (Salonia et al., 2020; Kerr et al., 2024).

Next, work by Agusti et al. presents a detailed review of what is known regarding citrus flowering. Citrus is a valuable perennial crop with a long juvenile period and comprises cultivars that vary greatly in their floral load. Here, the authors provide a comprehensive overview of factors influencing floral onset, including genes, environmental factors, and endogenous hormones, all of which represent possible means to alter floral timing. In these species, many genes are conserved with *Arabidopsis*. Moreover, citrus also contains interesting gene family expansions, including *FT*, which has three known citrus homologs. This paper ties in what is known about the molecular mechanisms of floral onset with treatments known to accelerate the flowering process.

In *Avena sativa* (oat), a current main floral challenge is that long days are strictly required for floral onset, which currently limits the geographic locations in which oats can be cultivated. In Zhang et al. the authors used gene expression studies of oats grown under both short- and long-day photoperiods to identify possible targets to alter floral onset in oat. They found that some major differences in gene expression involved pathways related to hormone regulation and the photoperiodic regulation of floral onset. Taken as a whole, this new data can be used to develop new cultivars of oat that are capable of flowering under shorter day lengths, thereby expanding the cultivation range of this important crop.

*Oryza sativa* (rice) is another globally valuable food staple. At present, there is considerable interest in improving the rice grain yield, and altering the structure of the inflorescence is one promising approach to achieve this goal. Here, a paper by Chun et al. reviews the current state of knowledge regarding the molecular genetics of rice inflorescence formation. For example, rice contains many genes that are conserved with *Arabidopsis*, and which are also found in other monocot species. However, grain monocots have many structural differences from dicots, and genes related to floral development have often taken on specialized functions, making them potential targets for influencing inflorescence structure. Finally, the authors also present a thoughtful commentary on the nuanced challenges associated with characterizing environmental influence on final rice yield in different environments.

Many plant species are used in horticulture, where their decorative features are highly prized. *Dahlia pinnata* Cav. (dahlias) are popular ornamental plants with showy compound blooms and many cultivars. While popular as a garden planting, dahlias have a limited usage as cut flowers due to accelerated senescence of the blooms. In this current paper, Casey et al. investigate gene expression analysis underlying senescence. Here again, *Arabidopsis* gene network data was used to aid in data interpretation, and the authors found that genes involved in hormone pathways were altered during bloom senescence. Next, by applying these findings they confirmed that treatment with cytokinin improved the longevity of cut flowers for different cultivars. However, outcomes of treatments with ethylene inhibitors varied among cultivars.

In a more specialized area, Shu et al. reviewed what is currently known in the field of bulbil formation. Bulbils are small, non-floral asexual reproductive structures that can develop on many regions of plants, including on what would have been an inflorescence. Understanding how and when bulbils form, and identifying key genes involved in the process of their formation, are important for plant cultivation and propagation. In this paper the authors also propose specific cultivation methods for increasing bulbil production that may lead to more reliable and predicable bulbil formation.

Taken together, we believe that these recent articles showcase some of the many ways in which learning more about the process of floral onset, structure and longevity can be applied to improve the performance of flowering plants, and that the application of this knowledge may lead to important breakthroughs in crop production, crop yield, horticulture, and in related fields.

## Author contributions

AK: Writing – original draft, Writing – review & editing. TE: Writing – original draft, Writing – review & editing. PH: Writing – original draft, Writing – review & editing.

## References

[B1] Causier B. Schwarz-Sommer Z. Davies B. (2010). Floral organ identity: 20 years of ABCs. Semin. Cell Dev. Biol. 21, 73–79. doi: 10.1016/j.semcdb.2009.10.005 19883777

[B2] Coen E. S. Meyerowitz E. M. (1991). The war of the worls: genetic interactions controlling flower development. Nature 353, 31–37. doi: 10.1038/353031a0 1715520

[B3] Kerr S. C. Shehnaz S. Paudel L. Manivannan M. S. Shaw L. M. Johnson A.. (2024). Advancing tree genomics to future proof next generation orchard production. Front. Plant Sci. 14. doi: 10.3389/fpls.2023.1321555 PMC10834703 38312357

[B4] Kim D.-H. (2020). Current understanding of flowering pathways in plants: focusing on the vernalization pathway in Arabidopsis and several vegetable crop plants. Horticulture Environment Biotechnol. 61, 209–227. doi: 10.1007/s13580-019-00218-5

[B5] Kobayashi Y. Kaya H. Goto K. Iwabuchi M. Araki T. (1999). A pair of related genes with antagonistic roles in mediating flowering signals. Science 286, 1960–1962. doi: 10.1126/science.286.5446.1960 10583960

[B6] Rehman S. Bahadur S. Xia W. (2023). An overview of floral regulatory genes in annual and perennial plants. Gene 885, 147699. doi: 10.1016/j.gene.2023.147699 37567454

[B7] Salonia F. Ciacciulli A. Poles L. Pappalardo H. D. La Malfa S. Licciardello C. (2020). New plant breeding techniques in citrus for the improvement of important agronomic traits. A Review. Front. Plant Sci. 11, 1234. doi: 10.3389/fpls.2020.01234 PMC7456868 32922420

